# Evisceration of appendix via drain site - a rare complication in a child

**DOI:** 10.1016/j.ijscr.2023.108111

**Published:** 2023-04-04

**Authors:** Ghulam Mustafa, Ali Asad, Imran Hashim, Nudrat Farooq, Chaudhary Abdul Fatir, Muhammad Sohaib Asghar

**Affiliations:** a Children Hospital, Lahore, Pakistan; b Services Hospital, Lahore, Pakistan; c Lahore General Hospital, Lahore 54000, Pakistan; d Dow University of Heslth Sciences, Ojha Campus, Karachi, Pakistan

**Keywords:** Drain, Complications, Herniation, Appendix, Evisceration, Case report

## Abstract

**Introduction and importance:**

Abdominal drains prevent fluid accumulation and help drain fluid (blood, pus). In recent years, abdominal drains have been used less frequently due to drain-associated complications, like infections, anastomotic leakage, and the potential for the evisceration of intra-abdominal organs on drain removal.

**Case presentation:**

We present a young female with evisceration of the appendix via the drain site, successfully managed by abdominal exploration and appendectomy.

**Case discussion:**

The use of prophylactic abdominal drain is controversial and is of limited use even in complicated appendicitis in the modern era of antibiotics. If a drain is inserted, it should be removed at the earliest to avoid associated complications.

**Conclusion:**

Abdominal drain usage should be minimized in children to avoid post-operative complications.

## Highlights

• Abdominal drains can occasionaly cause evisceration of intra-abdominal organs on drain removal.
• We present a young female with evisceration of the appendix via the drain site.
• Abdominal drain usage should be minimized in children to avoid post-operative complications.

## 1 Introduction

The use of post-surgical abdominal drains varies in clinical settings [1]. It becomes necessary in intestinal surgeries, especially the colon, because of potential fecal contamination of the peritoneal cavity and wound area. A drain mitigates the risk of intra-abdominal infection and serves as an early warning system for anastomotic complications [2], [3]. Drains can act as a source of infection, induce anastomotic leakage, and potentially eviscerate the intra-abdominal organs on drain withdrawal [3], [4]. The evisceration of abdominal organs, like intestinal loops or fallopian tubes, has been reported in adults but is rare in children. This report describes a rare abdominal drain complication in a child who underwent colo-colic anastomosis. The case report was reported according to the guidelines assembled by SCARE group [5].

## 2 Case report

**Fig. 1:**
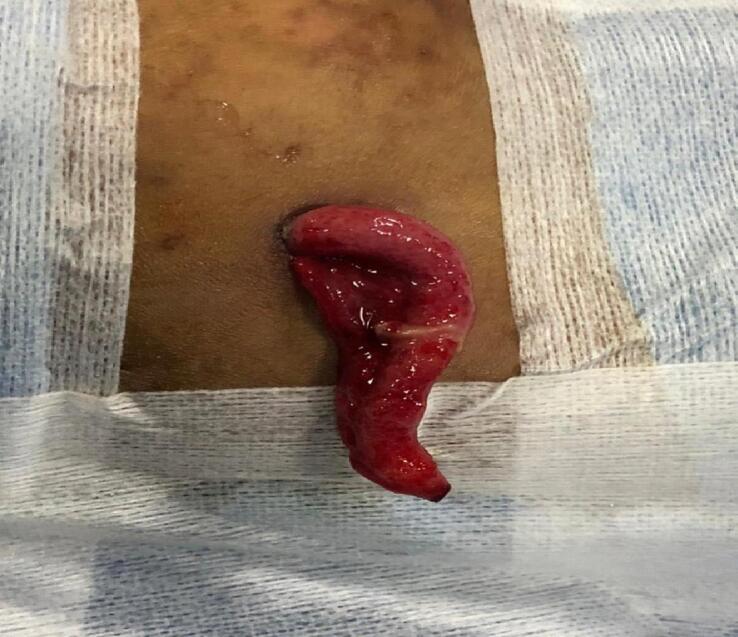
Clinical photograph showing eviscerated appendix.

**Fig. 2:**
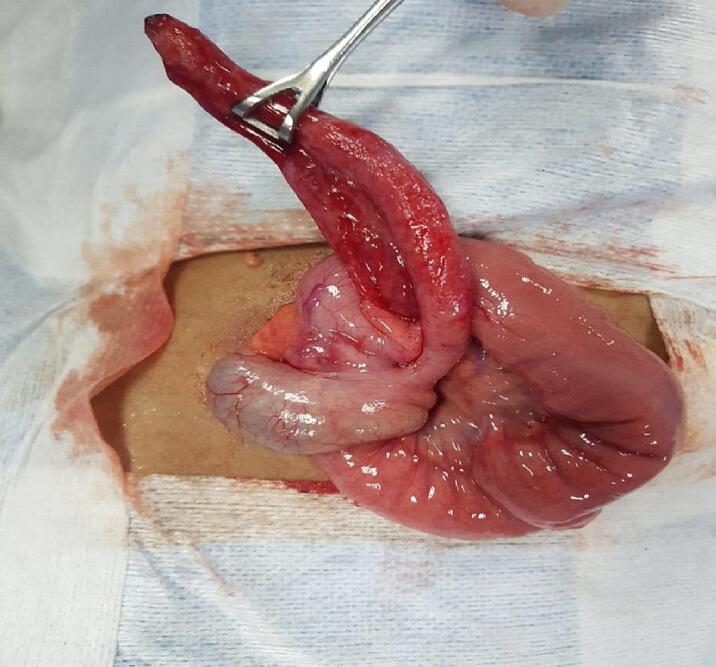
Operative photograph showing the ischaemic changes of appendix.

A 5-year-old female was admitted to the pediatric surgery ward for a colostomy reversal. Sigmoid loop colostomy was done six months back as a diversion stoma for a perianal injury involving the anal sphincter due to a road traffic accident. Colostomy reversal was done by the end-to-end colo-colic anastomosis under general anesthesia, and a 20-French Nelton drain was placed in the pelvis via the right flank. On the third post-op day, the patient started to pass stool via anal opening. On the fourth post-op day, the drain was removed, and with the drain, the vermiform appendix was eviscerated through the drain incision (Fig. 1). The patient was taken into the operation theatre, an appendectomy was performed by extending the previous drain incision, and the wound was closed (Fig. 2). Stoma closure was done from the same site with extension of incision to the pelvis (hockey shape incision) so that adhenolysis will be done. It made it easy to put drain from the right side of abdomen. The appendix was sent for histopathology. Post-operative recovery was uneventful, and the patient was discharged on the third post-op day after the appendectomy. Histopathology showed an inflamed appendix.

## 3 Discussion

Two types of drains are used in abdominal surgeries; open and closed. Open drains are corrugated rubber or plastic sheets, but their use is uncommon nowadays because of higher infection rates. Closed drains consist of tubes of inert material draining into a bag [6], [7]. Drain site evisceration is a rare complication of abdominal cavity drain. Several organs may eviscerate, including the omentum, small bowel, fallopian tube, gallbladder, and ovary [7], [8], [9], [10], [11], [12]. The mechanism of evisceration is not clearly understood. In adults, the predisposing factors include increased intra-abdominal pressure caused by coughing or straining, prolonged surgery, poor nutrition, wound infection, obesity, and steroid use [7]. In children, drain complications are rare. This may be because of less usage of drains or the small caliber of the drain. In our case, there was dense adhenolysis and chances of collection in pelvis, so it was decided on operating day to put a drain.

Riordan reported appendix drain site evisceration in 1995 [13]. A review of literature on PubMed using the Boolean operator strategy of “(appendix) AND (evisceration)” on 04/02/2022 yielded twelve results. This is probably the thirteen-reported case of appendix evisceration.

This complication is often noted immediately after removing the drain, but it may occur several hours or even a few days later [14]. Management varies from putting the appendix back into the abdominal cavity through an enlarged incision to appendectomy through the drain site, with or without hole enlargement [14]. Diameter of the drain is probably not the only factor influencing evisceration. The composition of the drain, its path through the abdominal wall, duration of drainage, pathology for which the patient is operated, and increased intra-abdominal pressure are factors to be considered [11]. The drains should be removed once the drainage has stopped.

The use of prophylactic abdominal drain is controversial and is of limited use even in complicated appendicitis in the modern era of antibiotics. Management varies from putting the appendix back into the abdominal cavity through an enlarged incision to appendectomy through the drain site, with or without hole enlargement as discussed in our case. As appendix looks inflamed near its tips, so it was decided to perform appendectomy.

## 4 Conclusion

Abdominal drain insertion should be avoided, especially in the pediatric population, unless necessary. If a drain is inserted, it should be removed at the earliest to avoid associated complications.

## Consent for publication

Written informed consent was obtained from the patient's guardian to publish this case report and any accompanying images. A copy of the written consent is available for review by the journal's Editor-in-Chief.

## Ethical approval

Ethical approval was obtained from institutional review board.

## Funding

There are no sources of funding.

## Author contribution

G.M., A.A., and I.H. conceived and designed the study, and were responsible for data collection and acquisition of data. G.M., A.A., N.F., C.A.F., and Z.Y. performed the literature review and wrote the manuscript. Z.Y., M.J.T., and M.S.A. reviewed and critically revised the manuscript. All authors have approved the final manuscript.

## Guarantor

Muhammad sohaib asghar

## Research registration number

Not applicable for case reports that does not deal a new surgical technique or new equipment/technology.

## Conflict of interest statement

There are no conflicts of interests.
